# Association between early childhood caries and parental education and the link to the sustainable development goal 4: a scoping review

**DOI:** 10.1186/s12903-024-04291-w

**Published:** 2024-05-02

**Authors:** Morenike Oluwatoyin Folayan, Elisa Maria Rosa de Barros Coelho, Imen Ayouni, Arthemon Nguweneza, Ola Barakat Al-Batayneh, Hamideh Daryanavard, Duangporn Duangthip, Ivy Guofang Sun, Arheiam Arheiam, Jorma I. Virtanen, Balgis Gaffar, Maha El Tantawi, Robert J Schroth, Carlos Alberto Feldens

**Affiliations:** 1 Early Childhood Caries Advocacy Group, Winnipeg, MB Canada; 2 Department of Child Dental Health, Obafemi Awolowo University, Ile-Ife, Nigeria; 3 Department of Pediatric Dentistry, Lutheran University of Brazil, Canoas, Brazil; 4 Department of Pediatric Dentistry, Pontifical Catholic University of Rio Grande do Sul, Porto Alegre, Brazil; 5 Department of Pediatrics and Child Health, Faculty of Health Sciences, University of Cape Town, Cape Town, South Africa; 6 Division of Human Genetics, Department of Pathology, Faculty of Health Sciences, University of Cape Town, Cape Town, South Africa; 7 Department of Preventive and Restorative Dentistry, College of Dental Medicine, University of Sharjah, Sharjah, United Arab Emirates; 8 Preventive Dentistry Department, Jordan University of Science and Technology, Irbid, Jordan; 9 Dubai Health Authority, Dubai, United Arab Emirates; 10 Faculty of Dentistry, The University of Hong Kong, Hong Kong SAR, China; 11 Department of Community and Preventive Dentistry, University of Benghazi, Benghazi, Libya; 12 Faculty of Medicine, University of Bergen, Bergen, Norway; 13 Department of Preventive Dental Sciences, College of Dentistry, Imam Abdulrahman bin Faisal University, Dammam, Saudi Arabia; 14 Department of Pediatric Dentistry and Dental Public Health, Faculty of Dentistry, Alexandria University, Alexandria, Egypt; 15 Dr. Gerald Niznick College of Dentistry, University of Manitoba, Winnipeg, Canada; 16 College of Dentistry, The Ohio State University, Columbus, OH USA

**Keywords:** Paternal education, Maternal education, Oral hygiene practices, Dental care, Feeding behaviors, Early childhood caries

## Abstract

**Background:**

The goal of the United Nations Sustainable Development Goal (SDG) 4 is to ensure inclusive and equitable quality education and promote lifelong learning opportunities for all. The aim of this scoping review was to map the current evidence on the association between the prevalence of early childhood caries (ECC) and parental education; and to identify possible pathways by which parental education may protect against ECC.

**Methods:**

The two questions that guided this review were: what is the existing evidence on the association between maternal and paternal education and ECC; and what are the pathways by which parental education protects against ECC? The initial search was conducted in January 2023 in PubMed, Web of Science and Scopus. Articles published in English between January 2000 and October 2022 that reported on the association between parental education and ECC were screened, and the extracted data were compiled, summarized, and synthesized. Review papers and non-primary quantitative research papers were excluded from the full-text review. Open coding was applied to develop a conceptual framework.

**Results:**

In total, 49 studies were included: 42 cross-sectional, 3 case-control and 4 cohort studies. The majority (91.8%) reported on the associations between ECC and maternal (*n* = 33), paternal (*n* = 3), and parental (*n* = 9) level of education, and 13 (26.7%) reported on the association between parental education and the severity of ECC. Mothers with more than primary school education (*n* = 3), post-secondary/college/tertiary education (*n* = 23), and more than 4–12 years of education (*n* = 12) had children with lower risk for ECC. Two studies reporting on parental education found an association between maternal but not paternal education and ECC. The review suggests that achieving the SDG 4.1 may reduce the risk of ECC. Possible pathways by which maternal education protects from ECC were feeding practices, oral hygiene practices, and the use of dental services.

**Conclusion:**

The study findings suggests that higher maternal educational level may reduce the risk for the consumption of cariogenic diet, poor oral hygiene practices and poor use of dental services for caries prevention. However, the association between paternal education and ECC was not consistently observed, with significant associations less frequently reported compared to maternal education. Future studies are needed to define the magnitude and modifiers of the impact of maternal education on the risk for ECC.

## Introduction

The goal of the United Nations’ Sustainable Development Goal (SDG) 4 is to ensure inclusive and equitable quality education and promote lifelong learning opportunities for all [1]. Access to education is critical as it is the principal pathway to financial security, stable employment, safe neighborhoods, healthier lifestyles, and social success, all of which protect or enhance health [2, 3]. Education also improves individuals’ knowledge, skills, reasoning, effectiveness, and other abilities that can be utilized to achieve optimal health [4]. Earning an education credential is a potent signal about one’s skills and abilities to be economically and socially secured [4]. Education influences health and longevity through a causal relationship that results in skills acquisition and the ability to be dynamic and flexible with mechanisms [5–10].

The health of young children is intrinsically linked to the educational level of their parents; education improves economic opportunities by reducing financial difficulties among households and improves health literacy [11]. The relationship between parental education and the health of the child is stronger for maternal than paternal education, however this varies by racial and ethnic background [12]. The exact mechanism through which parental education affects the health of the child health is still unclear [13, 14]. Low parental health literacy increased the risk of under-5 mortality [15], child malnutrition [14], unnecessary visits to emergency departments, mistakes in the administration of medication, as well as increased risk of exposure to secondhand tobacco smoke [13].

Parental education may also be intrinsically linked to the risk for early childhood caries (ECC), defined as cavitated and non-cavitated caries lesions present on the teeth of children < 72 months [16]. An analysis of the ECC chain of causality shows that socioeconomic factors are among the determinants of behavioral factors that cause the disease including the early introduction of sugar and high frequency of sugar consumption in the diet of infants, toddlers, and pre-school children [17–24] and other oral health behaviors [25]. Among non-communicable diseases, ECC is the first consequence of sugar consumption and can affect children even before completing the first year of life [18–20]. Parental level of education is associated with offering sugar in the first year of life [18, 26] and early introduction of mature oral bacteria [27] both of which are important factors associated with ECC. However, little is known about the pathways by which parental education may affect the risk for ECC. This information can inform public policies that address the SDG 4.

Parental education is intricately linked to SDG4, as it affects the health and wellbeing of communities. Education fosters health and healthy lifestyle choices, empowers individuals to adopt and sustain healthy behaviours throughout their lives, addresses health inequalities, and strengthens community resilience to health threats and emergencies [28]. The 10 targets and 11 indicators of the Sustainable Development Goal 4 are all focused on ensuring access to equitable and quality primary and secondary education that promotes effective learning outcomes [29].

Thus, the aim to this scoping review was to map the current evidence on the associations between parental (maternal and paternal) education and ECC, and to investigate possible pathways by which parental education may protect against ECC. The study also identified the link between studies on parental education and ECC with the SDG 4.

## Methods

This scoping review was reported according to the Preferred Reporting Items for Systematic Reviews and Meta-Analyses Extension for Scoping Reviews (PRISMA-ScR) guidelines [30].

### Research questions

The research question that guided this study was: What is the existing evidence on the association between maternal and paternal education and ECC? An additional question investigated was: what are the pathways by which maternal and paternal (parental) education protects against ECC?

### Identifying relevant articles

The initial search was conducted on three electronic databases in January 2023. PubMed, Web of Science and Scopus. The search was performed using the following key terms: “early childhood caries”, “dental caries”, “tooth decay” “parental”, “maternal”, “mother”, “father”, “paternal”, “education”, “schooling”. Search terms were tailored to the specific requirements of each database. Publications, including e-pub ahead from 2000 to 2023, were screened. Additional hand searching was done. The search was completed in January 2023. No protocol was published for this review.

### Eligibility and selection

Literature obtained through database searches were exported to the reference management software Zotero version 6, where duplicates were removed using the “duplicates items” function. Title and abstract screening were conducted by two independent reviewers, guided by eligibility criteria for this review. No authors or institutions were contacted to identify additional sources.

### Inclusion criteria

This review only included English language publications from January 2000 until October 2022. Studies that were cross-sectional, case-control, and cohort in design, and presented findings about the association between education and ECC among children below the age of six years were included.

### Exclusion criteria

As the aim of this review was to assess the association between parental education and ECC, studies focusing exclusively on ECC prevalence were excluded from this review. Ecological studies were excluded as well. Review papers and non-primary quantitative research papers were excluded from the full-text review screening and analysis.

### Data extraction

The data extraction was performed in four phases. The first phase was conducted by one reviewer (IA) who searched in the three databases for the information. In the second phase, the same reviewer screened the title and abstracts of all identified manuscripts and removed the duplicates. In the third phase two reviewers (IA and AN) reviewed the manuscripts independently and discrepancies were discussed with a third reviewer (MOF) to reach a consensus. The name of the author, publication year of the manuscript, study location, study design, study sample size, age of the children studied, study aim, data collection methods, and main findings were extracted from the studies included in this review. The extracted data from each study were compiled and summarized in Table 1. In the fourth phase, the summarized data was shared with two experts for their review (CAF and EMRBC). Publications were retained only when there was consensus between the experts and the earlier three reviewers. In the fifth phase, the consensus document was shared with members of the Early Childhood Caries Advocacy Group (www.eccagroup.org) to identify other published ECC literature reporting on the association between ECC and parental education not retrieved by the original search strategy.

### Synthesis of results

There were two steps for data synthesis. First, a descriptive analysis of the publications included in the review was conducted. The descriptions included countries where the studies were conducted, the study design, the journals (dental or non-dental) in which the studies were published and the results on the associations between maternal/paternal education levels and ECC. Countries where the studies were conducted were classified by World Health Organization region into Americas region (AMR); Eastern Mediterranean Region (EMR); African region (AFR), European region (EUR); Southeast Asian region (SEAR) and the Western Pacific region (WPR).

Next, open coding was applied for the identification of concepts, categories, or themes to generate initial codes that capture the main ideas emanating from the study findings [31]. The findings were used to revise the categories of the chart and establish key themes across the included manuscripts. From this analysis, a conceptual framework was created based on an analysis done using the socioecological model and revised by all authors. The developed conceptual framework could guide future empirical studies on the links between parental education and ECC. It can also guide policy making by identifying entry points for interventions and policies. For the current study, the conceptual framework illustrates how the exposure of interest (parental education) can directly or indirectly influence the outcome (ECC).

### Role of the funding source

There was no funding for the study. The study design selection, data collection, data analysis, data interpretation and writing of the report were free from any form of influence. All authors had full access to all the data in the study and had final responsibility for the decision to submit for publication.

## Results

**Fig. 1:**
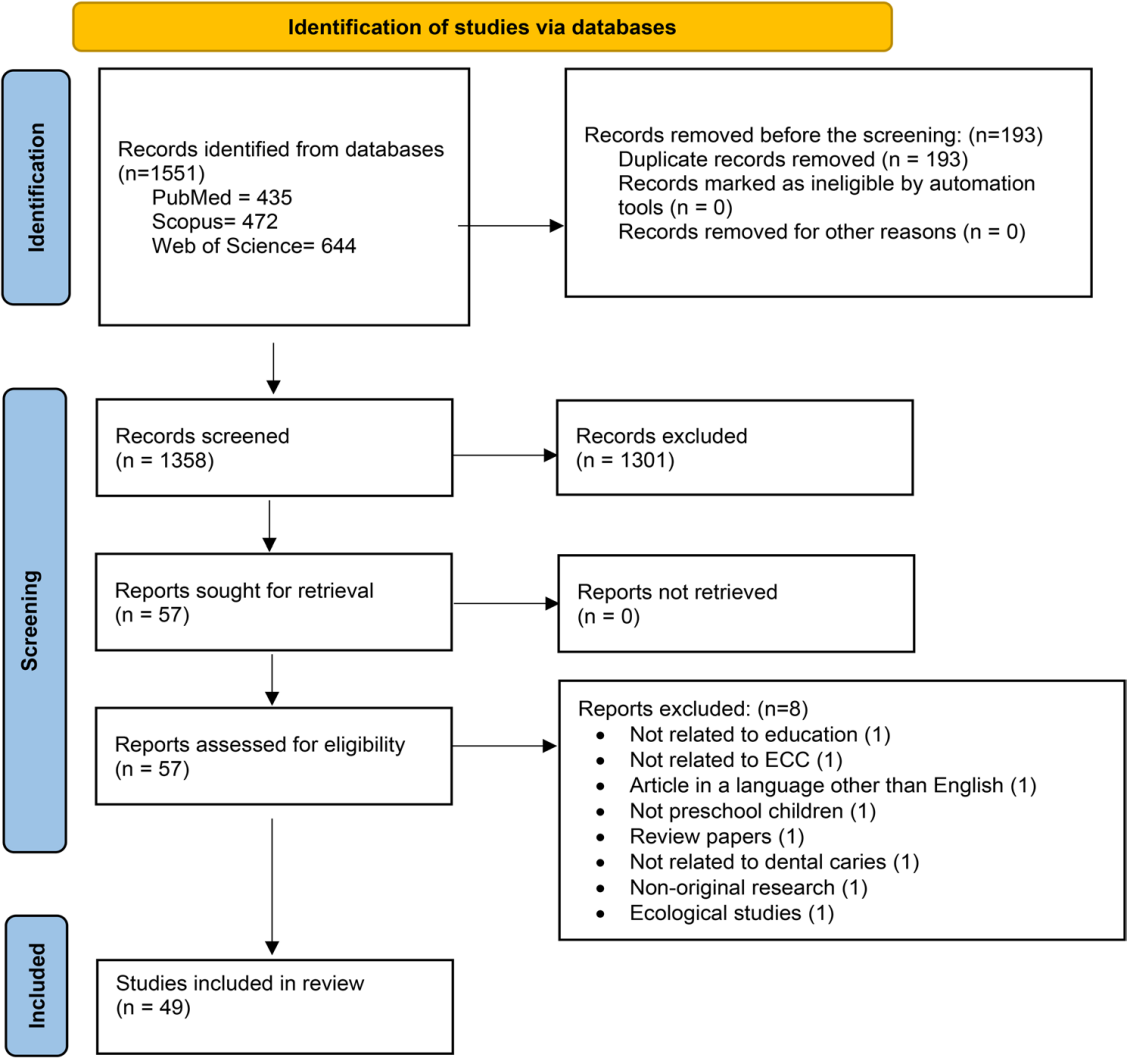
Study flowchart showing the flow of studies from retrieval to the final included studies

The initial search from PubMed, Web of Science and Scopus using the specified search terms yielded 1,551 potentially relevant articles. Of these, 193 papers were removed as duplicates, leaving 1,358 papers for title and abstract screening. Following that, 1,301 papers were excluded based on the eligibility criteria. Fifty-seven articles were eligible for full text screening. Studies that have ecological design or non-primary design, reported in a language other than English, did not assess the association between education related factors and ECC or had a sample of children older than six years were excluded. Finally, 49 studies that met the inclusion criteria were included in this scoping review [32–80]. All included articles assessed the association between maternal and/or paternal education and ECC. Figure 1 illustrates the flow of publication identification.

As shown in Table 1, the studies included in the review were conducted in all the World Health Organization regions: SEAR (*n* = 14), AMR (*n* = 11), WPR (*n* = 11), AFR (*n* = 4), EUR (*n* = 5), and EMR (*n* = 4). When split by continent, the majority of the studies were conducted in Asia (52.1%), leaded by China [33, 42, 43, 45, 46, 54, 62]., This was followed by South America (16.7%), leaded by Brazil [39, 41, 53, 55, 56, 59, 64, 71] and then followed by the Middle East (8.34%), Africa (8.3%) and Europe (8.3%). The least number of studies was conducted in North America (6.3%).

In addition, 42 (85.7%) studies were cross-sectional in design [32, 34–46, 48–53, 56–68, 70–75, 77, 78, 80], three were case-control [47, 54, 79] and four were cohort [33, 55, 69, 76] studies. In addition, 42 (85.7%) studies were published in dental journals [32, 33, 36–38, 41–48, 53–80] and seven (14.3%) studies were published in non-dental journals [34, 35, 39, 40, 49–51].

Of the 49 studies reviewed, 45 (91.8%) reported an association between maternal and or paternal education and the prevalence of ECC. These include 33 (73.3%) reports on maternal education [33, 35, 37, 39, 41–45, 47, 49–57, 59–61, 63, 67, 69, 71, 72, 74, 76–80], three (6.7%) reports on paternal education [34, 40, 62] and 9 (13.3%) reports on both fathers and mothers’ level of education [36, 38, 46, 48, 64, 65, 68, 70, 75] associations with ECC. Three (6.1%) studies did not find an association between ECC and maternal education [32, 58, 66], while one (2.0%) study did not find an association with paternal education [73]. In addition, two studies reported an association between maternal education and ECC but not with the father’s education [50, 57].

Of the 45 studies that identified an association between maternal and or paternal education and the prevalence of ECC, 13 (28.9%) identified and association between maternal [36, 45, 51, 56, 57, 66, 71, 72, 74, 78, 80], paternal [45] or parental [48, 62] level of education and the severity of ECC. In addition, different levels of maternal and or paternal education were associated with ECC.

The articles included in this scoping review that indicated an association between maternal and or paternal education and ECC suggest that achieving the SDG 4.1 – ensure that all girls and boys complete free, equitable and quality primary and secondary education - may reduce the risk of ECC at the family level in the future [81]. Table 1 summarizes the included studies.

**Table 1:** Summary of the 49 studies included in the scoping review

First author (year)	Country	Sample size/ age	Study design	ECC prevalence	Main findings
**African region**					
Abiola Adeniyi (2009) [58]	Nigeria	404 18–60 months	Cross-sectional	10.9%	Mother’s educational level was not associated with ECC.
Alade (2021) [66]	Nigeria	1549 6–71 months	Cross-sectional	4.3%	Mother’s educational level was not associated with ECC and its severity.
Masumo (2012) [32]	Tanzania Uganda	122 Tanzania 816 Uganda 6–36 months	Cross-sectional	3.7% in Tanzania 17.6% in Uganda	Maternal and paternal educational level were not associated with ECC.
Kabil (2017) [68]	Egypt	140 2–4 years	Cross-sectional	64.2%	The children whose mothers and fathers had a university education had lower odds of ECC than those whose mother’s and father’s education was limited to lower than university education.
**Region of the Americas**					
Schroth (2005) [78]	Canada	61 3 years	Cross-sectional	44%	The severity of ECC was lower as the mother’s educational status increased from ‘did not finish high school’ to ‘completed high school’ and ‘post-secondary education’.
Foxman (2022) [76]	USA	650 3 years	Cohort	6%	Children of mothers with educational level lower than high school presented with a higher prevalence of ECC.
Finlayson (2007) [52]	USA	719 1–5 years	Cross-sectional	54%	Children of mothers with high school or more educational level had lower risk for ECC.
Ferreira (2007) [53]	Brazil	1487 0–5 years	Cross-sectional	40%	Mother’s education < 4 years associated with greater probability of ECC.
Oliveira (2008) [59]	Brazil	1018 12–59 months	Cross-sectional	23.4%	Children whose mothers had < 8 years of education had an increased risk of high levels of dental caries.
Traebert (2009) [64]	Brazil	347 3–5 years	Cross-sectional	64.3%	Children of mothers with < 8 years of education had higher prevalence and severity of ECC.
Feldens (2010) [55]	Brazil	340 48–53 months	Cohort	63%	Mother’s education ≤ 8 years was associated the occurrence of S-ECC at 4 years of age.
Carvalho (2014) [71]	Brazil	2511 1–5 years	Cross-sectional	39.5%	The likelihood of ECC was higher for children of mothers with 4 years of education and < 4 years of education.
Kramer (2014) [56]	Brazil	2,793 < 6 years	Cross-sectional	39.6% in 2000 and 25.9% in 2010	Children whose mothers had *≤* 8 years of education had higher caries prevalence and dmft scores than those whose mothers had > 8 years of education. Significant reduction in dmft from 2000 to 2010 only in children of mothers with > 8 years of education.
Pinto-Sarmento (2016) [39]	Brazil	843 36–71 months	Cross-sectional	66.3%	Mother’s schooling ≤ 8 years was significantly associated with higher odds of ECC.
Pinto (2017) [41]	Brazil	538 24–42 months	Cross-sectional	15.1%	Maternal education level (≤ 8 years or > 8 years) was not associated with the prevalence of caries in children.
**Eastern Mediterranean region**					
Sayegh (2002) [63]	Jordan	1140 4–5 years	Cross-sectional	67%	Children of mothers with intermediate college and university education had lower ECC prevalence.
Elamin (2018) [57]	UAE	186 18–48 months	Cross-sectional	41%	Children of mothers with university education had significantly lower ECC severity and SiC index score than children of mothers with high school education and below. The father’s educational level was not associated with ECC severity and SiC index score.
Yazdani (2020) [47]	Iran	500 4–6 years	Case-control	NA	Children of mothers and fathers with university education were less likely to have ECC.
Jamshidi (2022) [75]	Iran	280 3–5 years	Cross-sectional	73.2%	Parental education was not associated with ECC.
**European region**					
Declerck (2008) [65]	Belgium	2533 3–5 years	Cross-sectional	7% for 3-year-olds and 31% for 5-year-olds	Children whose mothers and fathers had complete college/higher/university education had a lower risk of developing dental caries than those whose mothers and fathers had completed primary/secondary school.
Congiu (2014) [37]	Italy	544 18–60 months	Cross-sectional	15.9%	Lower odds of ECC in children from mothers and fathers with secondary school and university degree compared to primary school.
Olczak-Kowalczyk (2020) [48]	Poland	656 3 years	Cross-sectional	53.8%	Children of parents with higher education level had significantly lower odds of having ECC, severe-ECC and had lower ECC severity.
Hernandez (2021) [72]	France	425 4 years	Cross-sectional	15.8%	Children with mothers whose education level was high school or lower had higher prevalence of ECC and higher dmft than those whose mothers’ education level was university. Father’s education was not associated with dmft.
Ersin (2006) [80]	Turkey	101 15–35 months	Cross-sectional	9%	Higher dmft in children with mothers with < 4years educational level.
**South-East Asia region**					
Jigjid (2009) [61]	Mongolia	670 1–5 years	Cross-sectional	72.2%	The caries severity was significantly higher in children with mothers with university and higher educational level than children with high school and lower educational level.
Nanayakkara (2013) [34]	Sri Lanka	784 48–72 months	Cross-sectional	72%	ECC was significantly higher in children whose fathers had ≤ 5 years of education compared to those whose fathers had tertiary education.
Vachirarojpisan (2004) [60]	Thailand	520 6–19 months	Cross-sectional	82.8%	Children of mothers with educational level of primary school or less has higher I- ECC values than those whose mothers’ education level was secondary school or higher.
Peltzer (2015) [69]	Thailand	597 3 years	Cohort	68.5%	Children with mothers who had primary and high school had significantly higher odds of having S-ECC than children of mothers who had no education at the time of birth of the children.
Boonyawong (2022) [50]	Thailand	338 4–5 years	Cross-sectional	80.8%	Mother’s educational level and not fathers’ educational level was significantly associated with ECC. Children of mothers who had mandatory education had higher odds of having ECC than children of mothers with higher education.
Agarwal (2011) [79]	India	150 3–5 years	Case-control	NA	Higher prevalence of ECC in children from mothers with education up to intermediate.
Prakash (2012) [73]	India	1500 8–48 months	Cross-sectional	27.5%	The prevalence of caries was significantly higher in children with mothers who had no schooling and primary education alone than those who had higher secondary school, pre-university college, graduate and postgraduate education.
Narang (2013) [35]	India	512 2–6 years	Cross-sectional	33%	The prevalence of ECC was significantly lower as the maternal educational status increase from being illiterate to having primary, high school and university education.
Sankeshwari (2013) [36]	India	1116 3–5 years	Cross-sectional	63.2%	Inversely correlation between mother’s education and dmft.
Stephen (2015) [70]	India	2771 18–72 months	Cross-sectional	16%	The prevalence of ECC was high among children with illiterate parents and was lowest among children with parental education at college level.
Bhayade (2016) [38]	India	324 1–5 years	Cross-sectional	63.6%	Significantly more children whose parents had education level less than school site council had ECC.
Vandana (2018) [44]	India	550 2–6 years	Cross-sectional	-	The mother’s educational level at child’s birth and not the father’s educational level at child’s birth was significantly associated with the prevalence of ECC.
Nagarajappa (2020) [77]	India	320 3–6 years	Cross-sectional	37.2%	Children whose mother had no schooling had significantly higher prevalence of caries prevalence.
Chhabra (2022) [51]	India	398 4–5 years	Cross-sectional	45.2%	The prevalence and severity of ECC reduced as the educational level of the mother increased from below 10th grade to 10th grade pass, to graduate education and postgraduate education.
**Western Pacific region**					
Qin (2008) [54]	China	246 0–4 years	Case-control	NA	The proportion of children that were caries free increased from mothers with elementary education to mothers with high school education to mothers with college education and higher.
Li (2011) [62]	China	1523 3–6 years	Cross-sectional	71.8%	The prevalence of caries was significantly higher in children whose parents had elementary education than those who had middle school or college education. dmft decreased from elementary to college educational level of parents.
Zhou (2012) [33]	China	225 8–32 months	Cohort	28.4%	Children whose mother had ≥ 12 years of education at the time of birth of the child had significantly lower odds for ECC.
Li (2017) [43]	China	1727 3–5 years	Cross-sectional	78.2%	Children with mothers who has educational level higher than none/primary education had significantly higher odds of lower ECC prevalence.
Sun (2017) [42]	China	337 24–37 months	Cross-sectional	9.8%	Higher mothers’ education level was associated with higher possibility of ECC experience.
Li (2020) [46]	China	2592 3–5 years	Cross-sectional	78.4%	Children whose parents had education of more than 9 years had significantly lower odds of having ECC than children with parents who had 9 years or less of education.
Kato (2017) [40]	Japan	6315 3 years	Cross-sectional	14.7%	Compared with less than 13 years of paternal and maternal education, 13–14 years and 15 or more years of paternal and maternal education were independently inversely significantly associated with the prevalence of ECC.
Gao (2018) [67]	Hong Kong	5167 3 years	Cross-sectional	22%	Significantly more children whose mother and father had higher than mandatory education had ECC when compared with mothers and fathers who had the mandatory education respectively.
Duangthip (2019) [45]	Hong Kong	1204 3–5 years	Cross-sectional	46.3%	Significantly fewer children with mothers and fathers that had tertiary education and above had ECC. The severity of ECC was also lower was significantly lower for children with mothers and fathers that had tertiary education than children with mothers and fathers who had secondary, primary and below primary education.
Zheng (2021) [49]	Hong Kong	404 5 years	Cross-sectional	57%	Significantly more children of mothers with secondary school or below had untreated ECC and decayed teeth than children of mothers with post-secondary school education.
Nguyen (2018) [74]	Vietnam	1028 2–5 years	Cross-sectional	89.1%	The ECC prevalence and severity in children aged 2-years and 3-5-years were significantly lower in children whose mother had high school education and above than those with up to middle school.

Table 2 shows the possible pathways by which parental education may protect against ECC. The three mediating variables identified were all related to maternal education. The first was feeding practices: earlier introduction of sugary foods and drinks and higher risk of cariogenic feeding practices in children of mothers’ lower maternal education [82–84]. Second, higher maternal education was associated with better oral hygiene index, less plaque and Strep mutans count, and earlier introduction of fluoride toothpaste [58, 80, 85–87]. Third, higher level of maternal education was associated with the use of dental services and a higher proportion of dental visitation by children [88–90].

**Table 2:** Mechanisms explaining how parental education level may protect against ECC

First author (Year)	Country	Study design	Sample size	Main findings
**Feeding practices**				
Feldens (2012) [55]	Brazil	Cohort	327	The risk of cariogenic feeding practices doubled in children from mothers with less < 5 years of education.
Tovar (2019) [84]	USA	Cohort	666	Children of mothers with less than high-school degree when compared with those with mothers with high-school degree or higher were introduced early to juice and sugar-sweetened-beverage consumption.
Feldens (2021) [83]	Brazil	Cohort	596	A larger number of sweet items introduced in the first year of life in children whose mothers were < 8 years of schooling.
**Hygiene practices/biofilm/fluoride dentifrice use**				
Ersin (2006) [80]	Turkey	Cross-sectional	110	Mother’s education < 4 years indirectly associated with ECC through the Strep mutans count.
Adeniyi (2009) [58]	Nigeria	Cross-sectional	404	Maternal education was positively correlated with children oral hygiene index.
Feldens (2010b) [85]	Brazil	Cross-sectional	432	Maternal education > 4 years was associated with the introduction of fluoride- dentifrice before 2 years of age.
Duijster (2014) [86]	Netherlands	Cross-sectional	630	Lower maternal education level was related to factors associated with poorer oral hygiene behaviours, higher levels of ECC.
Azevedo (2015) [87]	Brazil	Cross-sectional	249	Children whose maternal schooling was < 8years had higher risk for dental plaque than children whose mothers had > 8years education.
**Use of dental services**				
Machry (2013) [88]	Brazil	Cross-sectional	478	Lower prevalence of dental visitation among children whose parents had low socioeconomic backgrounds and who rated their oral health as poor.
Feldens (2018) [89]	Brazil	Cohort	435	Higher proportion of dental visitation in children whose mothers had more than > 8 years of education.
Folayan (2020) [90]	Nigeria	Cross-sectional	1549	Maternal education was not significantly associated with child’s dental-service utilization.

**Fig. 2:**
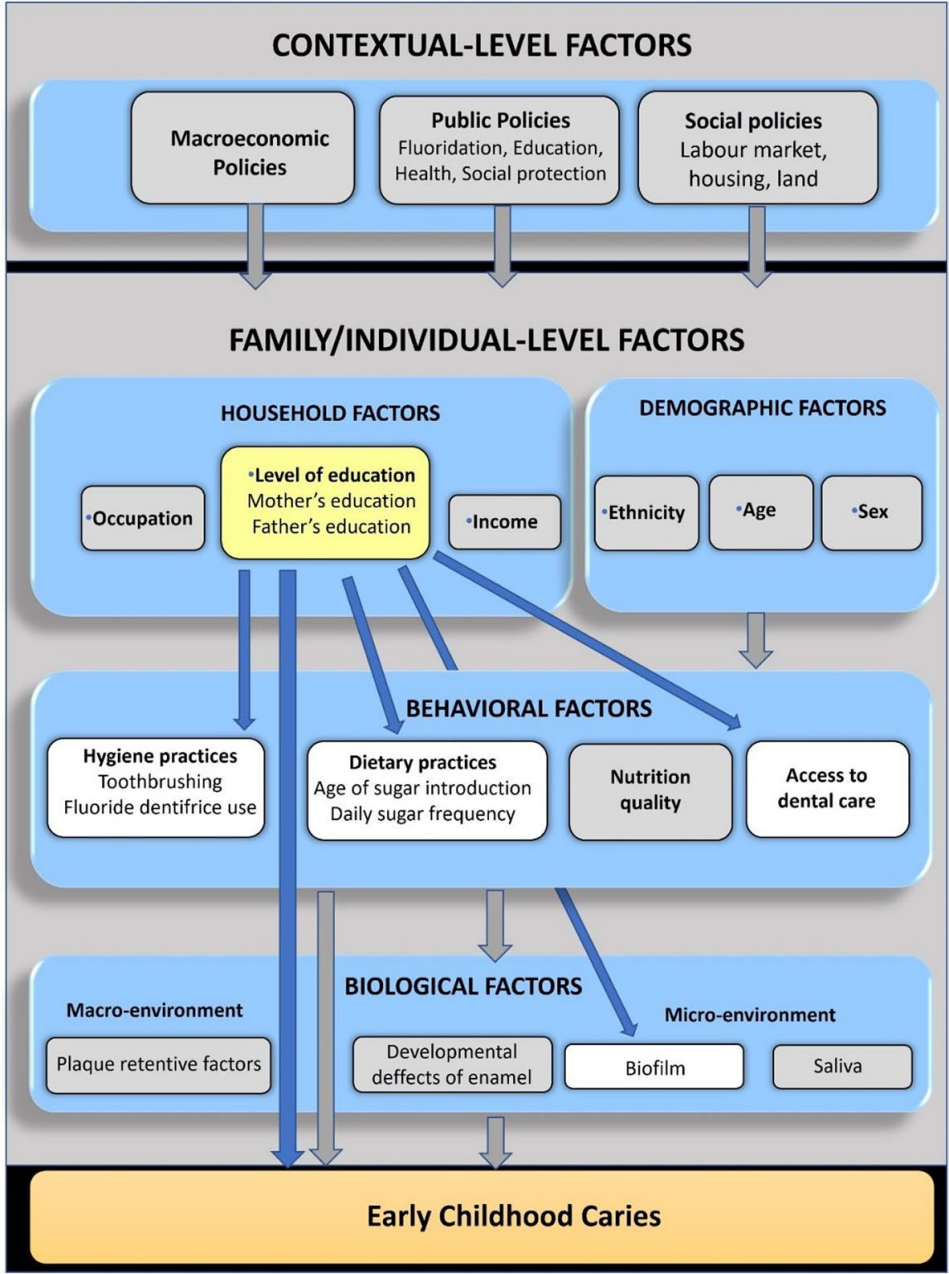
Conceptual framework depicting the relationships between contextual and individual factors and ECC. Blue arrows show the interactions between the variables analyzed in this study

Figure 2 presents the conceptual model that situates maternal and or paternal education as a risk factor for ECC using the socioecological model. The findings from the scoping review suggest a household factor (parental education) influences the behavioral and biological risk of infants, toddlers, and preschool children for caries.

## Discussion

The current evidence suggests that there is an association between maternal education level and ECC: the prevalence of ECC may be inversely related to the level of maternal education. There are however a few other insights revealed from our scoping review of the data.

First, the study findings suggest that parental education levels lower than 4 to 13 years were associated with higher risk for ECC while tertiary education is protective against ECC. Prior studies have indicated that adults’ access to tertiary education critically influences children’s general health, and lower levels in tertiary education adversely affect a country’s health situation [91]. This present review provides additional evidence to suggest that parental access to education above primary level of education – including maternal access to tertiary education – may lower the risk for ECC.

Second, not only did we find studies suggesting an inverse relationship between the prevalence of ECC and mothers’ educational level, but we also found studies suggesting an inverse relationship between the severity of ECC and mothers’ educational levels. A prior study had indicated that low maternal education increases the odds for treatment of ECC in the operating room using general anesthesia [92]. This suggests that low maternal education is associated with severe ECC as severe ECC is what requires treatment under general anesthesia [93]. Severe ECC affects general health and often causes pain, eating and sleeping difficulties, infection, impairs quality of life of affected children, results in absenteeism from school, and adversely affects the growth and development of the child [94]. It is therefore likely that regions of the world where poor attention is paid to maternal education, the risk of ECC may be high. Greater attention may, therefore, need to be paid to countries where the education of girls is given low priority in the attempt to reduce the global burden of ECC.

A region of interest is Africa, where the education of females is threatened by many factors such as conflict, economic deprivation, gender inequality, violence, traditional misconceptions, and social norms such as early marriage [95]. Africa is one of two regions of the world (the second is South-East Asia) with the highest burden of oral diseases [96]. The three studies that showed no associations between ECC and maternal education were conducted in countries in Africa [32, 58, 66]. A possible reason for these findings may be the high likelihood that the educational status of mothers largely cluster in the low educational status tercile. It is also possible that there are other drivers of the risk of ECC in the region beyond maternal education. Studies are, therefore, needed to unravel the role, impact, and pathways of influence of maternal and or paternal education on the risk of ECC.

We found that ECC risk behaviors such as feeding practices, hygiene practices and the use of dental services seem to be driven by the level of education of the mother. Children of mothers with low education tend to consume more cariogenic diets [82–84], use dental services less for preventive care [88, 89] and have poorer oral hygiene [58, 86, 87]. These are known risk factor for caries [97]. Thus, the findings from this scoping review presuppose that healthier diets, better hygiene practices, and increased use of services may be possible pathways by which parental education protects against ECC. Testing this hypothesis requires further investigation. There was, however, a study that indicated that maternal educational status may not be associated with dental service utilization [90].

Of interest was the observation in the current study that there was more evidence of an inverse association between the prevalence of ECC and paternal educational level than the evidence that showed no association. This is unlike most studies that report no significant associations between paternal education and the child’s health [98]. Prior studies had indicated that fathers with higher education use resources more efficiently, improve their access to financial capital, have a larger social networking, better communication skills, and healthier behaviors [82, 99]. These possibilities may have contributed to the lower risk of ECC. This postulation needs to be explored further.

Third, though there is a direct relationship between educational level and socioeconomic status, and socioeconomic encompasses income and educational attainment among others [100], the results of one of the studies suggested that education and not income was associated with the experience of ECC [33]. Some other maternal-related factors such as health beliefs, dental health locus of control, executive dysfunction, sense of coherence, dental self-efficacy, family organization, and access to social support may lead to better attitudes and behaviors and may explain the protective effect of mother’s education on childhood diseases [86, 101–103]. These factors need to be explored in future studies.

The exact mechanism by which parental education links with ECC is complex and needs to be deepened. The reviewed studies suggest that higher maternal education protects against ECC through lower sugar consumption, later introduction of sucrose, better oral hygiene practices, and use of dental services. This relationship, however, does not seem to be result from a simple “increase in knowledge” provided by greater education. Rather, mothers with higher education are likely to be more aware of the risk of their children developing ECC, positively appraise the benefits of preventive actions, and have a lower sense of fatalism [104]. Psychosocial variables like perceived susceptibility, perceived severity, perceived benefits, and perceived barriers as represented by the Health Belief Model [105, 106], possibly represent a pathway by which higher maternal education protects against ECC through different mechanisms. In addition, the Extended Health Belief Model, used to examine the role of self-efficacy for other health problems [107–110], can be used to examine the role of maternal self-efficacy for the prevention of ECC. Maternal education may also affect fatalistic health beliefs, inadequate knowledge of children’s needs, the prospect of living in deprived neighborhoods [52, 111] and parenting style [112] all of which may increase the risk for ECC. The Extended Health Belief Model could also be combined with the Socioecological Model to understand contextual factors that directly or indirectly affects access of mothers and or fathers to education. Future studies on the link between parental education and ECC using theoretical models may help with identifying other parental related risk factors for ECC.

The Socioecological Model for the study of oral health in children [113] may also facilitate the study of other SDG 4 target’s direct and indirect influences on the risk of ECC. The current study on ECC and the SDG 4 suggests that the articles available on ECC is linked to only SDG 4.1. Thus, not only do we need a more comprehensive model developed to investigate factors that put children at risk of ECC using the parental education pathway, but models are also needed to enable us to learn how to ensure inclusive, equitable quality education and lifelong learning opportunities can reduce the risk for ECC.

The current study’s findings suggest several practical implications. Firstly, ensuring universal access to education and facilitating the educational progression of prospective mothers can potentially alleviate the burden of ECC. Additionally, there’s a call for policy implementations aimed at curbing teenage pregnancy, which is a pervasive factor contributing to school dropout rates and various adverse health outcomes throughout individuals’ live [114]. . Regarding future research directions, a systematic review with meta-analysis is needed to quantify the association between maternal/paternal educational level and the prevalence and severity of ECC. Such analysis could help identify regional and sub-regional level variabilities and provide information on the possible cutoff points at which the exposure variable (maternal/paternal education) significantly increases the risk for ECC. The systematic review and meta-analysis can also explore the role of contextual factors (fluoride in water, availability of health services) as a modifier of the effect of maternal and paternal educational level on the risk for ECC.

Despite the findings presented in this scoping review, a few limitations were identified. First, our search was confined to English-language literature, potentially excluding studies on the association between ECC and parental education published in other languages. This language restriction was strictly enforced during the article selection process for full-text review, ensuring transparency regarding the availability of eligible reports in languages other than English, as illustrated in Fig. 1 [115]. The decision to restrict our search to English literature was due to our inability to understand and interpret literature written in other languages. Second, our search was limited to three databases, which may have resulted in the omission of relevant articles not captured by the search strategy, possibly introducing selection bias. Despite these limitations, the study highlights plausible connections between ECC and parental education that warrant further empirical exploration in future research endeavours.

In addition, the current study was limited in its scope of definition of parents with no clear definition of the scope of parents – parents as biological parents or parents as caregivers. While biological parents typically refer to individuals who have a genetic relationship with the child, caregivers encompass a broader category of individuals responsible for providing care and support to children, including biological parents, adoptive parents, foster parents, grandparents, guardians, and other family members or non-family members entrusted with caregiving responsibilities. There are acknowledged complex distinctions on the impact of these roles on the health and wellbeing of children [116]. Understanding how these distinctions may affect the risk for ECC is crucial for comprehensively managing ECC.

## Conclusion

The findings of this scoping review showed that higher maternal educational level protects against ECC, with lower consumption of cariogenic diet and better oral hygiene practices being possible mediators of this relationship. However, a link between paternal level of education and ECC was not consistently observed, with significant associations less frequently reported compared to maternal education. The link between parental educational level and the risk for ECC may be moderated by multiple contextual factors suggesting the need for more studies from regions of the world with low publication rate – Africa, Europe, and the Eastern Mediterranean Regions. Studies on how other SDG 4 targets may influence the risk for ECC are also needed.

## Data Availability

The datasets used and/or analysed for the study are publicly accessible. Data used are summarised in the publication.

## Abbreviations

ECC: Early Childhood Caries
PRISMA-ScR: Preferred Reporting Items for Systematic Reviews and Meta-Analyses Extension for Scoping Reviews guidelines
SDG: Sustainable Development Goal
AMR: Americas region
EMR: Eastern Mediterranean Region
AFR: African region
EUR: European region
SEAR: South East Asian region
WPR: Western Pacific region
